# Supplementary material for: A single cell atlas of frozen shoulder capsule identifies features associated with inflammatory fibrosis resolution
Source: Nat Commun. 2024 Feb 19;15:1394. doi: 10.1038/s41467-024-45341-9 (PMC10876649; doi:10.1038/s41467-024-45341-9)
Supplement: Supplementary file 1 — Supplementary Information [file 41467_2024_45341_MOESM1_ESM.pdf]

A single cell atlas of frozen shoulder capsule identifies features associated with inflammatory fibrosis resolution

Supplementary Figures and Tables

Figure S1

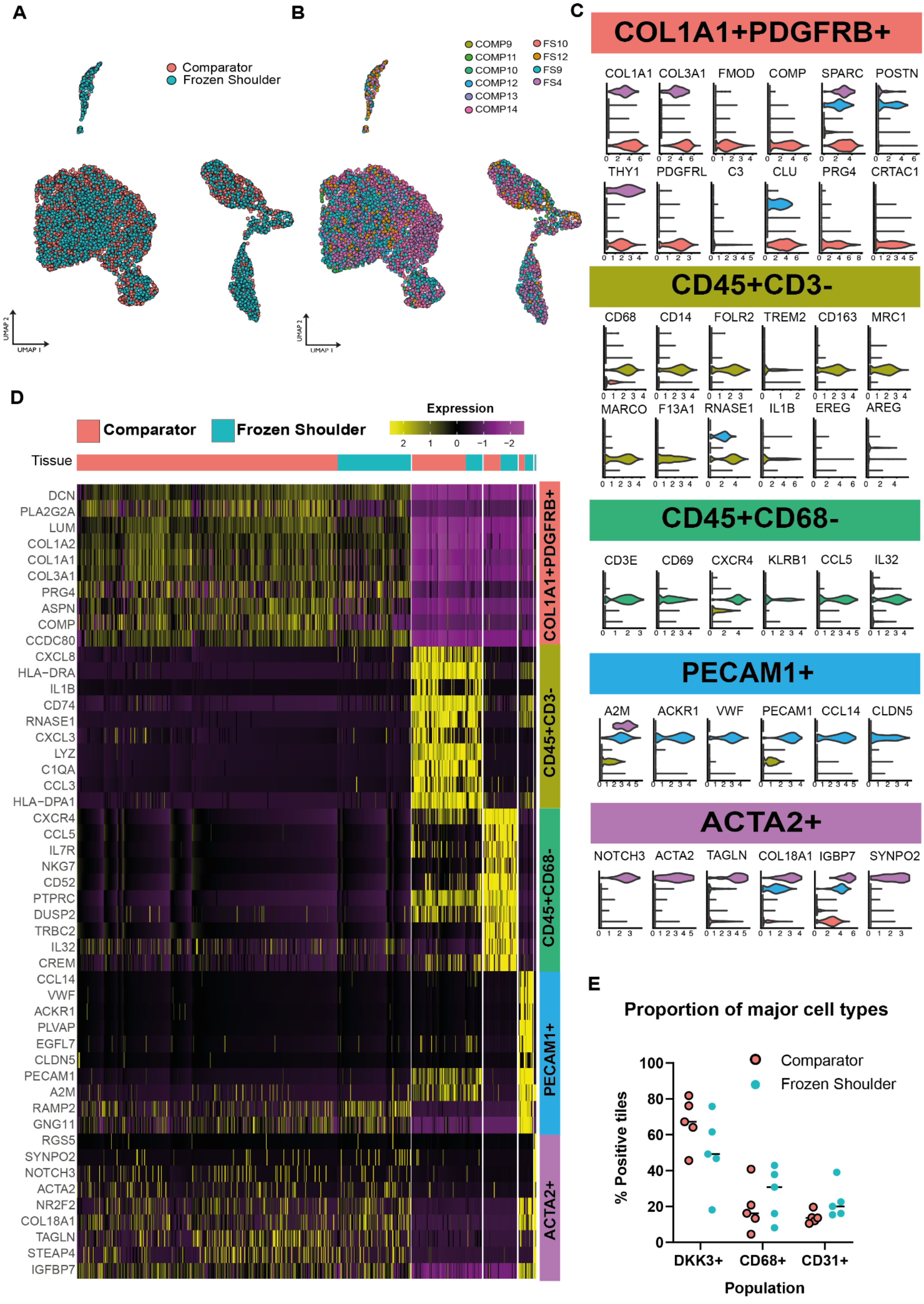
A
B
C
Comparator
Frozen Shoulder
UMAP 2
UMAP 1
COMP9
COMP11
COMP10
COMP12
COMP13
COMP14
FS10
FS12
FS9
FS4
COL1A1+PDGFRB+
COL1A1 COL3A1 FMOD COMP SPARC POSTN
THY1 PDGFRL C3 CLU PRG4 CRTAC1
CD45+CD3-
CD68 CD14 FOLR2 TREM2 CD163 MRC1
MARCO F13A1 RNASE1 IL1B EREG AREG
CD45+CD68-
CD3E CD69 CXCR4 KLRB1 CCL5 IL32
PECAM1+
A2M ACKR1 VWF PECAM1 CCL14 CLDN5
ACTA2+
NOTCH3 ACTA2 TAGLN COL18A1 IGBP7 SYNPO2
D
Comparator
Frozen Shoulder
Expression
2 1 0 −1 −2
Tissue
DCN
PLA2G2A
LUM
COL1A2
COL1A1
COL3A1
PRG4
ASPN
COMP
CCDC80
CXCL8
HLA−DRA
IL1B
CD74
RNASE1
CXCL3
LYZ
C1QA
CCL3
HLA−DPA1
CXCR4
CCL5
IL7R
NKG7
CD52
PTPRC
DUSP2
TRBC2
IL32
CREM
CCL14
VWF
ACKR1
PLVAP
EGFL7
CLDN5
PECAM1
A2M
RAMP2
GNG11
RGS5
SYNPO2
NOTCH3
ACTA2
NR2F2
COL18A1
TAGLN
STEAP4
IGFBP7
COL1A1+PDGFRB+
CD45+CD3-
CD45+CD68-
PECAM1+
ACTA2+
E
Proportion of major cell types
Comparator
Frozen Shoulder
% Positive tiles
100
80
60
40
20
0
DKK3+
CD68+
CD31+
Population

S1. Supplementary data for the major cell types found to be present in the adult shoulder capsule by single-cell RNA-sequencing.

- **(A)** UMAP of all cells present in the adult shoulder capsule (as shown in Figure 1B) coloured by condition.
- **(B)** UMAP of all cells present in the adult shoulder capsule (as shown in Figure 1B) coloured by sample.
- **(C)** The heatmap shows the top 10 conserved marker genes identified for each of the major cell populations comprising the adult shoulder capsule (as shown in Figure 1B). All identified cluster marker genes are provided in Supplementary Data 2.
- **(D)** Violin plots representing log-normalized expression values of selected marker genes of major clusters identified in comparator and frozen shoulder patient tissues including fibroblast (COL1A1+PDGFRB+), myeloid (CD45+CD3-), T cell (CD45+CD68-), vascular endothelial cell (PECAM1+) and mural cells (ACTA2+).
- **(E)** Graph shows protein validation for the relative proportions of major cell types comprising the shoulder joint capsule from comparator and frozen shoulder patient tissues in collective lining and sub-lining regions. Quantitative analysis of immunostained tissues was performed to support major populations including fibroblasts (DKK3+ cells), macrophages (CD68+ cells) and endothelial cells (CD31+ cells). Bar shows median values.

Figure S2

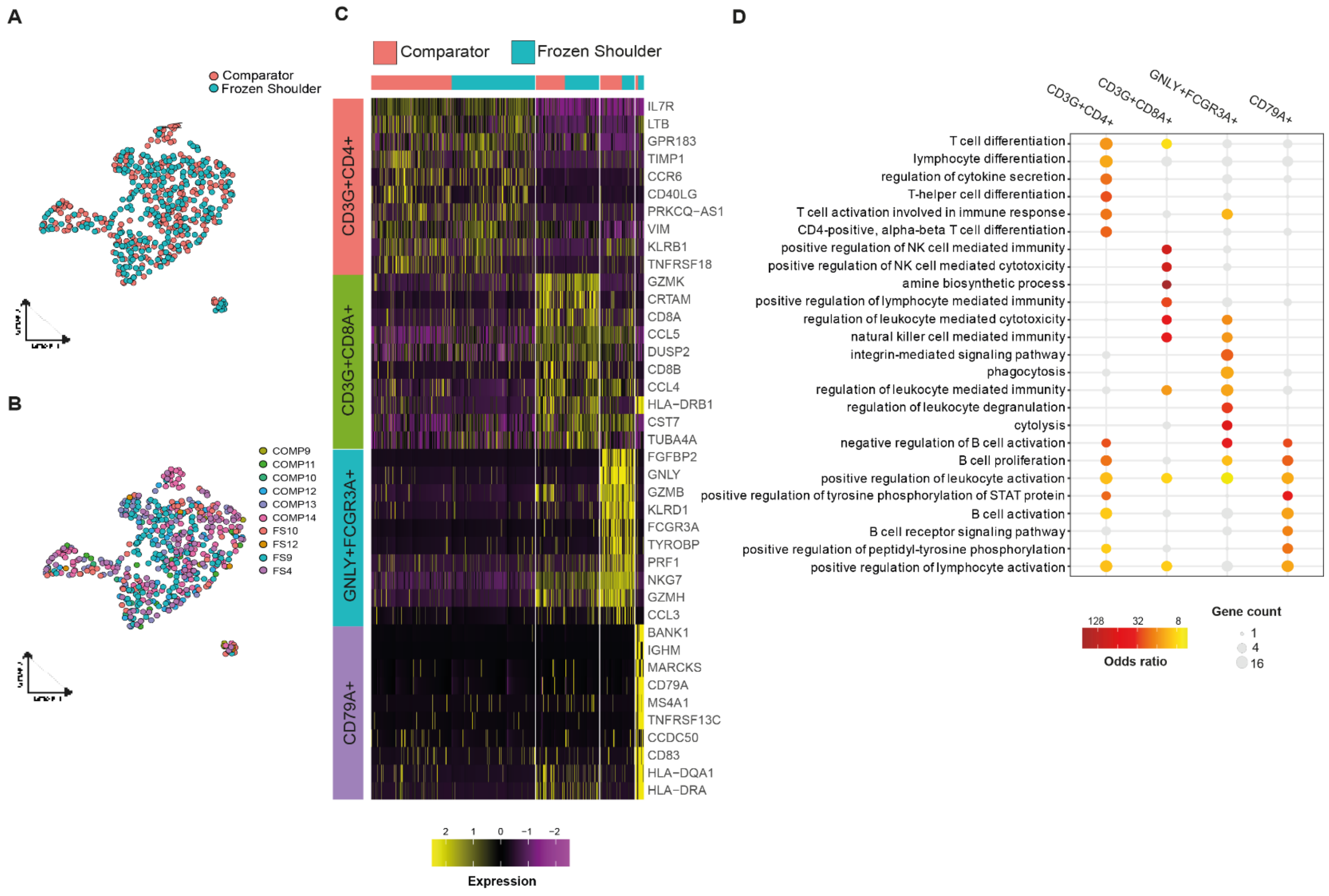

S2. Supplementary data for lymphoid populations found to be present in the adult shoulder capsule by single-cell RNA-sequencing.

- **(A)** UMAP of lymphoid cells present in the adult shoulder capsule (as shown in Figure 1D) coloured by condition.
- **(B)** UMAP of lymphoid cells present in the adult shoulder capsule (as shown in Figure 1D) coloured by sample.
- **(C)** The heatmap shows the top 10 conserved marker genes for each of the lymphoid clusters (Figure 1D, E). All of the identified cluster markers are provided in Supplementary Data 2.
- **(D)** The dot plot shows selected gene ontology (GO) biological processes found to be significantly over-represented in the lymphoid cluster marker genes (one-sided Fisher tests; BH adjusted *P* values < 0.05). All of the over-represented gene sets are provided in Supplementary Data 2.

Figure S3

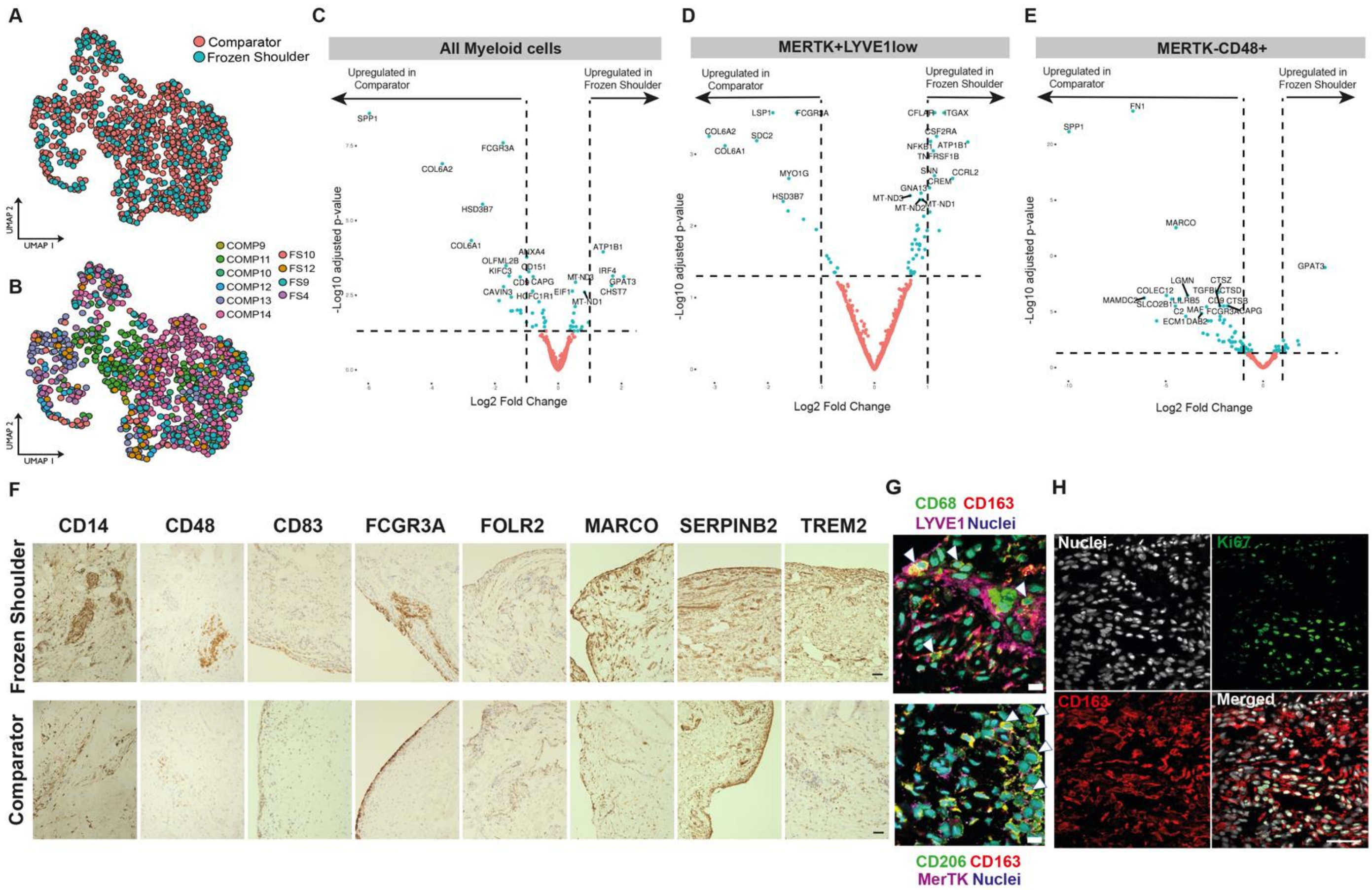

S3. Supplementary data for myeloid populations found to be present in the adult shoulder capsule by single-cell RNA-sequencing

- **(A)** UMAP of myeloid cells present in the adult shoulder capsule (as shown in Figure 1G) coloured by condition.
- **(B)** UMAP of myeloid cells present in the adult shoulder capsule (as shown in Figure 1G) coloured by sample.
- **(C)** The Volcano plot shows genes found to be differentially expressed between frozen shoulder and comparator patient tissues in "all myeloid cell" pseudobulks (DESeq2, Wald test). Significant genes (BH adjusted *P* <0.01) are shown in blue. See also Supplementary Data 3.
- **(D)** The Volcano plot shows genes found to be differentially expressed between frozen shoulder and comparator patient tissues in MERTK+LYVE1^{low} cluster pseudobulks (DESeq2, Wald test). Significant genes (BH adjusted *P*<0.01) are shown in blue. See also Supplementary Data 3.
- **(E)** The Volcano plot shows genes found to be differentially expressed between frozen shoulder and comparator patient tissues in MERTK-CD48+ cluster pseudobulks (DESeq2, Wald test). Significant genes (BH adjusted *P* <0.001) are shown in blue. See also Supplementary Data 3.
- **(F)** Representative bright field images show 3,3′-diaminobenzidine immunostaining (brown) immunostaining for key myeloid markers in comparator and frozen shoulder patient tissue sections. Nuclear counterstain is hematoxylin. Scale bar=50μm.
- **(G)** Representative confocal images show immunostaining for macrophage markers CD68, CD163 and LYVE1 in (top) and MRC1, CD163 and MerTK (bottom), white arrowheads highlight triple positive cells. Cyan represents POPO-1 nuclear counterstain, scale bars=10μm.
- **(H)** Panel shows representative confocal images of immunostaining for Ki67 (green) and CD163 (red) confirming the presence of proliferating macrophages in the capsular lining region of frozen shoulder patient tissues. Grey represents POPO-1 nuclear counterstain, scale bar=20μm.

Figure S4

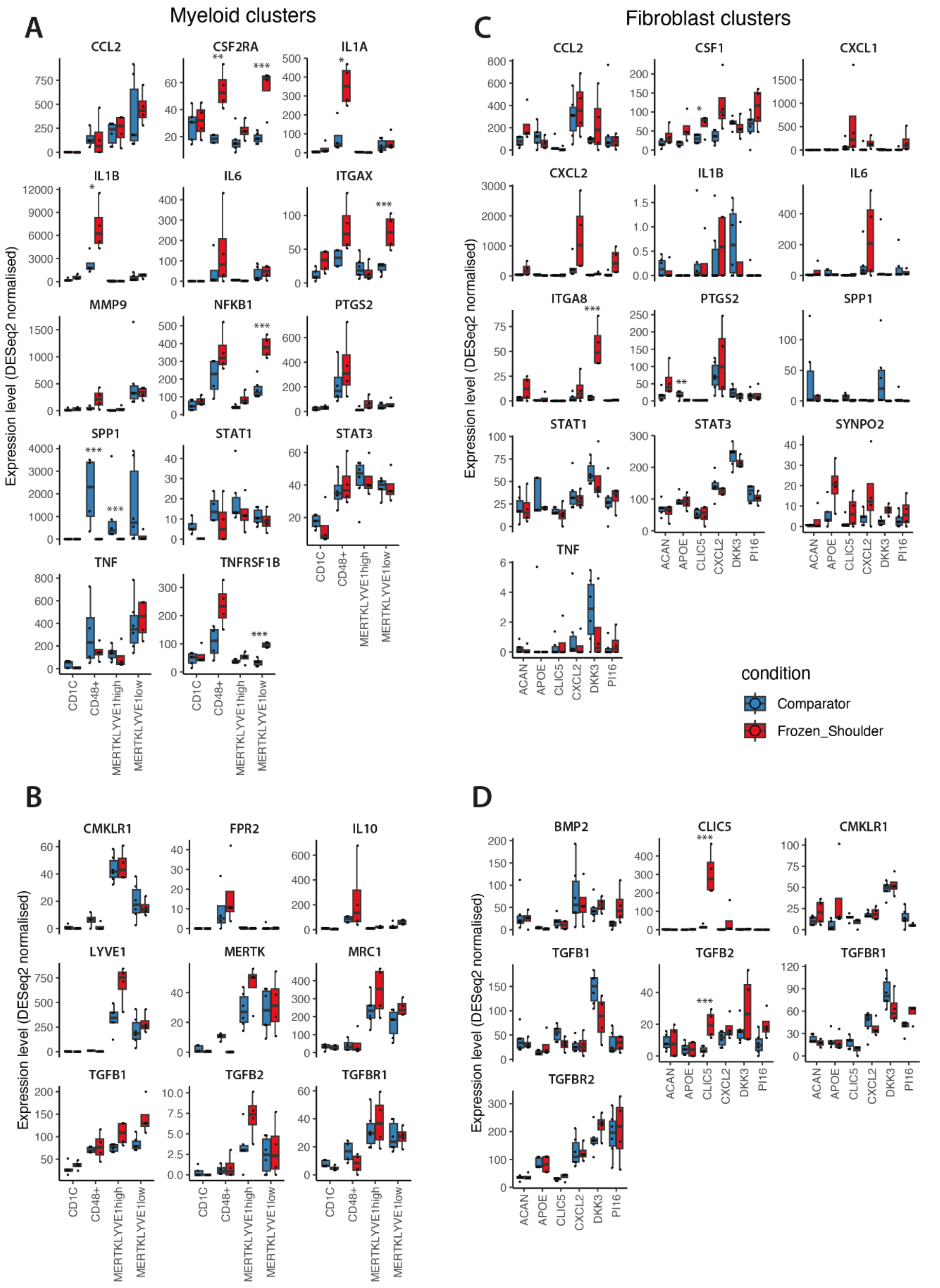

Figure S4. Expression of pro-inflammatory and immunomodulatory genes in human shoulder capsule tissues. Box plots show expression of **(A)** proinflammatory and **(B)** immunomodulatory genes in myeloid populations isolated from frozen shoulder (red, n=4 donors) and comparator (blue n=6) patient tissues (from Figure 1G). See also Supplementary Data 3. Box plots show expression of **(C)** proinflammatory and **(D)** immunomodulatory genes in fibroblast populations isolated from frozen shoulder (red, n=4) and comparator (blue n=6) patient tissues (from Figure 1J). See also Supplementary Data 4. Bar shows median values, ***P<0.001, **P<0.01, *P<0.05.

Figure S5

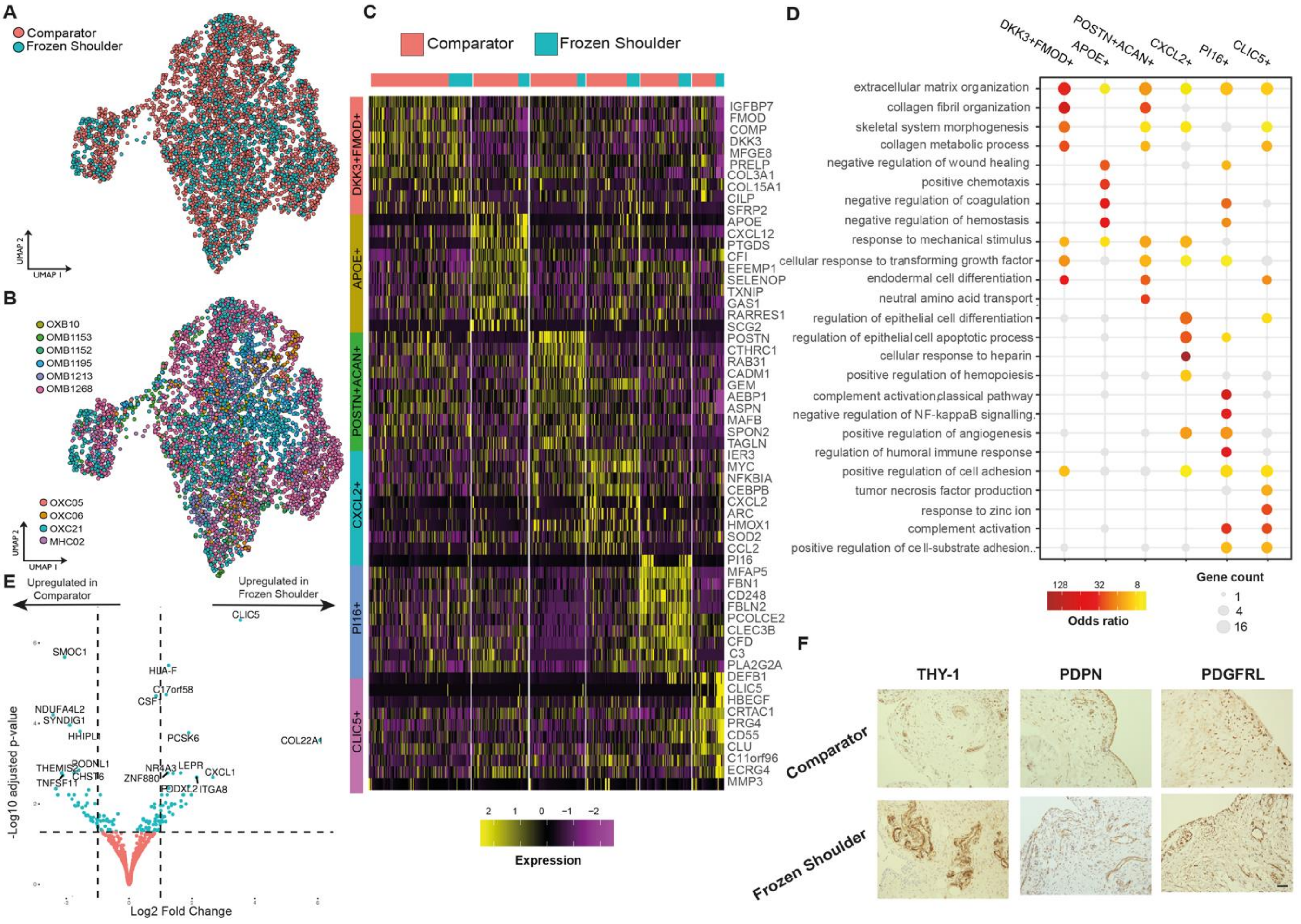

S5. Supplementary data for fibroblast populations found to be present in the adult shoulder capsule by single-cell RNA-sequencing

- **(A)** UMAP of fibroblast populations present in the adult shoulder capsule (as shown in Figure 1J) coloured by condition.
- **(B)** UMAP of fibroblast populations present in the adult shoulder capsule (as shown in Figure 1J) coloured by tissue.
- **(C)** The heatmap shows the top 10 conserved marker genes for each of the fibroblast clusters (Figure 1J). All of the identified cluster markers are provided in Supplementary Data 4.
- **(D)** The dot plot shows selected gene ontology (GO) biological processes found to be significantly over-represented in the fibroblast cluster marker genes (one-sided Fisher tests; BH adjusted *P* values <0.05). All of the over-represented gene sets are provided in Supplementary Data 4.
- **(E)** The Volcano plot shows genes found to be differentially expressed between frozen shoulder and comparator patient tissues in "all fibroblast cell" pseudobulks (DESeq2, Wald test). Significant genes (BH adjusted *P* <0.01) are shown in blue. See also Supplementary Data 4.
- **(F)** Representative bright field images show 3,3′-diaminobenzidine immunostaining (brown) immunostaining for fibroblast markers PDPN, THY1 & PDGFRL in comparator and frozen shoulder patient tissue sections. Nuclear counterstain is hematoxylin. Scale bar=50μm.

Figure S6

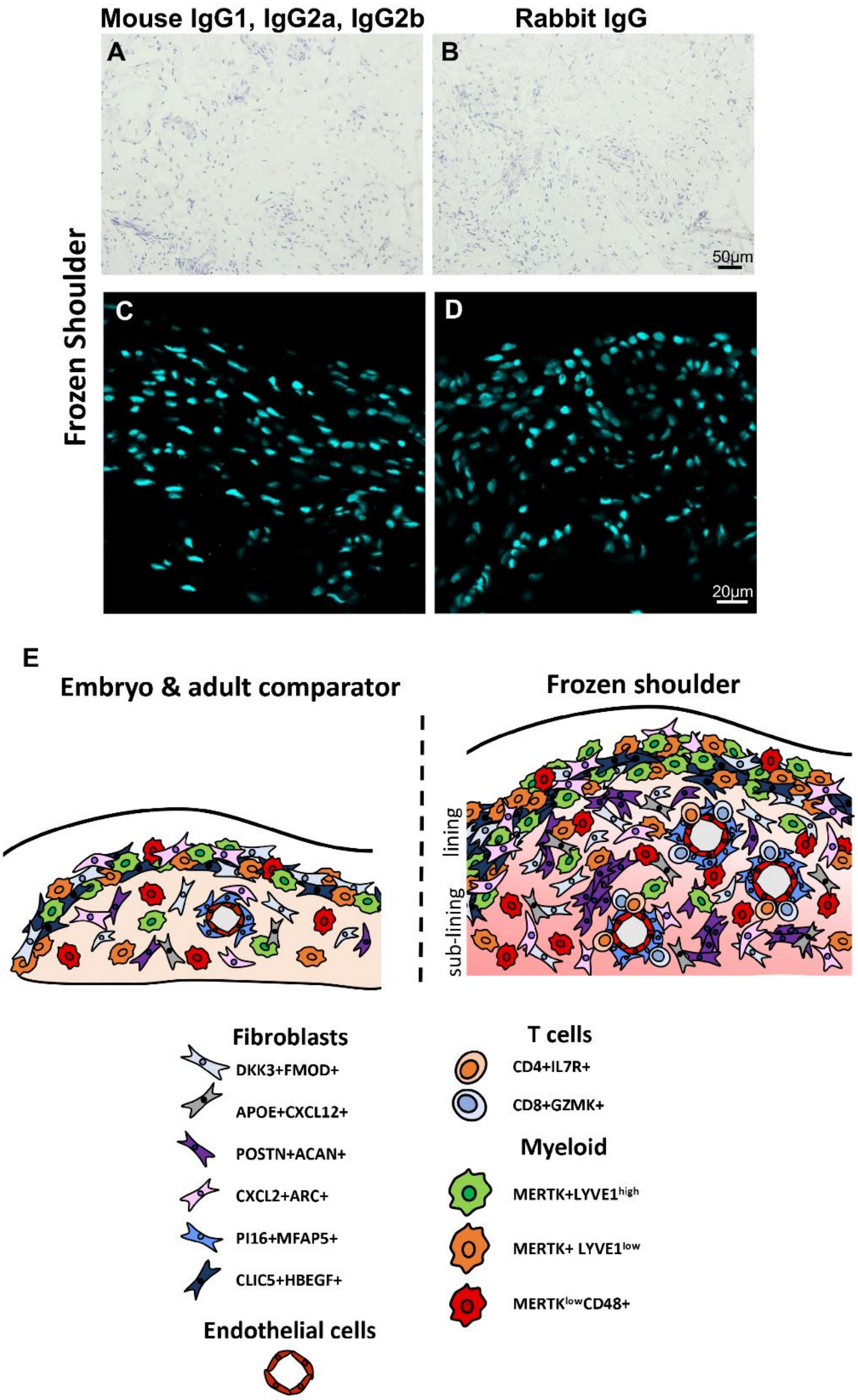

S6. Isotype control immunostaining of adult shoulder capsule tissues & schematic summarising the cellular and anatomical basis for resolving inflammatory fibrosis in the shoulder capsule.

(A&B) Panel shows representative bright field images of frozen shoulder tissue sections stained with isotype control antibodies for **(A)** mouse IgG1, IgG2a, IgG2b and **(B)** rabbit IgG fractions. Nuclear counterstain is haematoxylin. Scale bar=50μm.

(C&D) Representative confocal immunofluorescence images showing merged image of frozen shoulder tissue sections stained with isotype control antibodies for mouse IgG1, IgG2a, IgG2b **(C)** and rabbit IgG fractions **(D)**. Cyan represents POPO-1 nuclear counterstain. Scale bar=20μm.

(E) Schematic summarises the cellular and anatomical basis for resolving inflammatory fibrosis in the shoulder capsule. Macrophages and fibroblasts are comprised of distinct clusters, occupying specific microanatomical niches in embryonic, adult comparator and frozen shoulder capsule tissues. MERTK+ macrophages and CLIC5+ fibroblasts predominate in the capsule lining region, which is expanded during frozen shoulder. Frozen shoulder patient tissues show increased vascularity and enrichment for T cells relative to adult comparator tissues. T cell populations populate the capsule sub-lining adjacent to blood vessels. Frozen shoulder patient tissues are enriched for pro-inflammatory CD48+ macrophages which reside in capsule lining and sub-lining regions. POSTN+ACAN+ & PI16+MFAP5+ fibroblasts predominate in the capsule sub-lining adjacent to vascular endothelial cells, FMOD+DKK3+, APOE+CXCL12+ & CXCL2+ARC+ fibroblasts occupy lining and sub-lining regions.

Figure S7

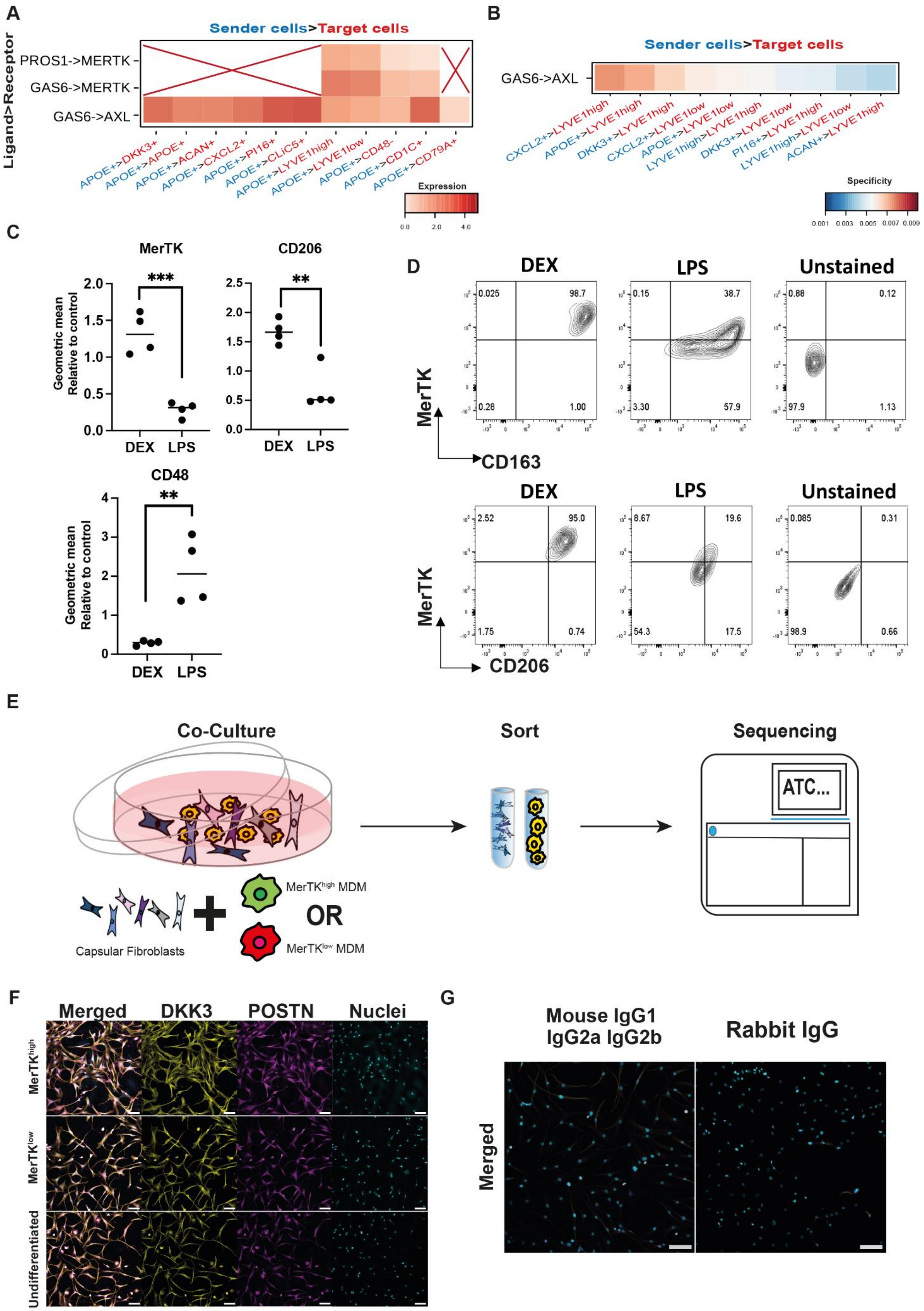
A
Sender cells>Target cells
Ligand>Receptor
PROS1->MERTK
GAS6->MERTK
GAS6->AXL
Expression
B
GAS6->AXL
Specificity
C
MerTK
CD206
CD48
Geometric mean Relative to control
DEX
LPS
D
DEX
LPS
Unstained
MerTK
CD163
CD206
E
Co-Culture
Sort
Sequencing
ATC...
Capsular Fibroblasts
MerTKhigh MDM
OR
MerTKlow MDM
F
Merged
DKK3
POSTN
Nuclei
MerTKhigh
MerTKlow
Undifferentiated
G
Mouse IgG1 IgG2a IgG2b
Rabbit IgG
Merged

S7. Capsular MerTK^{high} macrophages have a modulatory phenotype & characterisation of MerTK^{low} and MerTK^{high} MDMs by FACS

(A) NATMI analysis shows product between expression of sender ligand (PROS1 or GAS6) and receiver receptor on fibroblast, myeloid and lymphoid populations, cell types as in Figure 1.

(B) NATMI analysis shows product between specificity of sender ligand GAS6 and receiver receptor AXL is restricted to predicted fibroblast-fibroblast interactions, cell types as in Figure 1.

(C) Graphs show expression of macrophage markers MERTK, CD206 and CD48 in dexamethasone and LPS treated MDMs, indicating effects of treatments on macrophage phenotype relative to unstained cells. Data are derived from n=4 blood cone donors, bars show median values. Statistical analyses were performed using a t-test, ** P<0.01, ***P<0.001.

(D) Representative fluorescence-activated cell sorting (FACS) contour plots from monocyte derived macrophages (MDMs) treated with either dexamethasone (1µM) or LPS (10ng/ml) to induce MerTK^{high} and MerTK^{low} macrophage phenotypes respectively. Staining is shown relative to unstained cells.

(E) Schematic highlights co-culture experiments whereby capsular fibroblasts (n=3 frozen shoulder donors) were directly co-cultured with MerTK^{high} or MerTK^{low} MDMs (MDMs isolated from 3 blood cones) in 2 independent experiments.

(F) Representative confocal images show immunocytochemistry staining for fibroblasts isolated from frozen shoulder patients co-incubated in MerTK^{high}, MerTK^{low} or undifferentiated monocyte-derived macrophages. Markers of matrix associated fibroblasts include DKK3 (yellow) & POSTN (magenta), supporting the presence of the matrix-associated fibroblasts that are predicted to interact with MerTK+ macrophages. Experiments performed using capsular fibroblasts isolated from n=3 frozen shoulder donors. Cyan represents nuclear counterstain, scale bar = 100µm.

(G) Representative confocal immunofluorescence images showing merged image of capsular fibroblasts from frozen shoulder patients co-incubated with monocyte-derived macrophages stained with isotype control antibodies for mouse IgG1, IgG2a, IgG2b and rabbit IgG fractions. Cyan represents POPO-1 nuclear counterstain, scale bar=100µm.

Figure S8

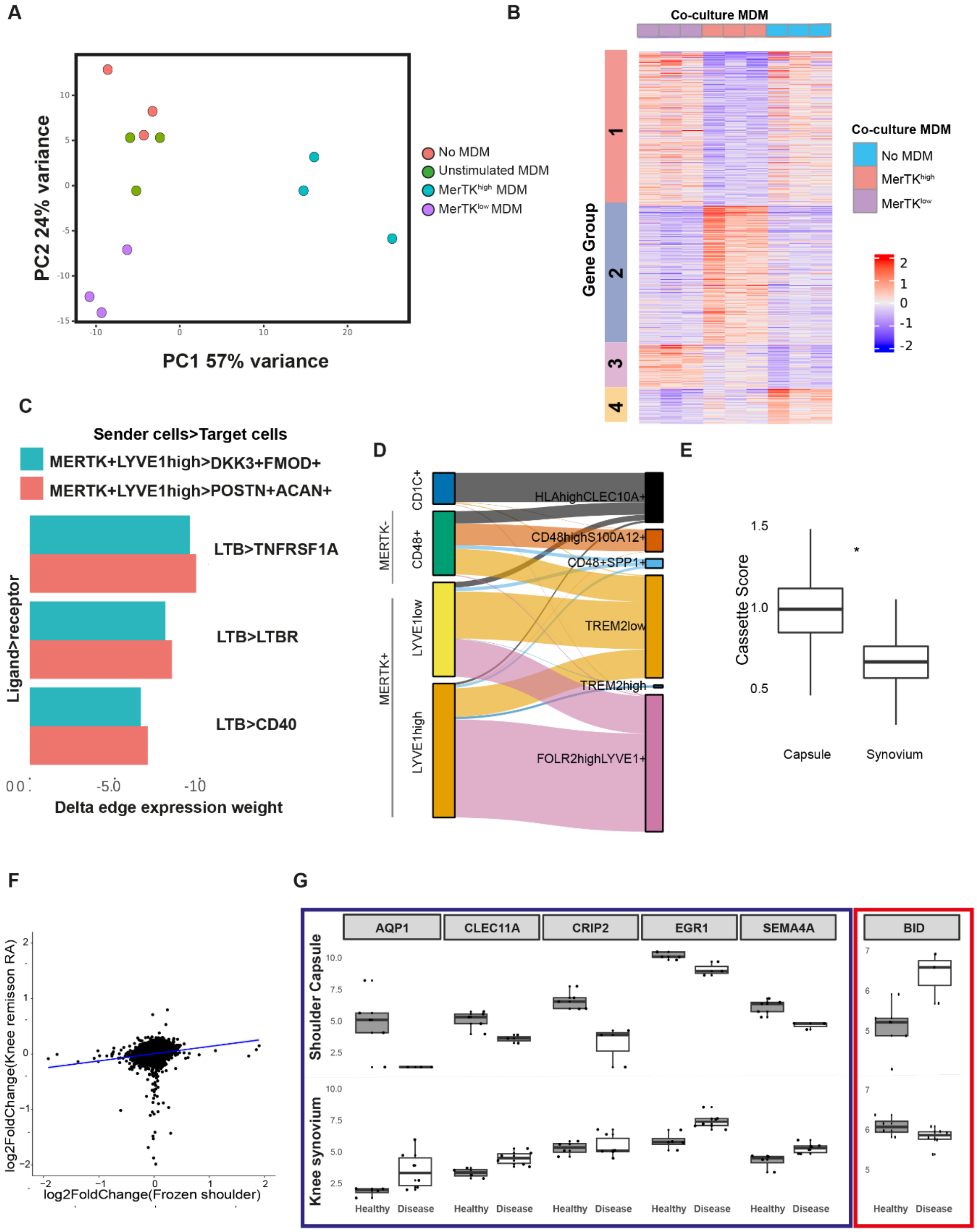

S8. Comparison of MERTK^{high} macrophage clusters in shoulder capsule and RA synovial tissues.

(A) The scatter plot shows transcriptional variation between (i) untreated capsular fibroblasts (red, “no MDM”), (ii) capsular fibroblasts co-cultured with unstimulated monocyte-derived macrophages (MDM) (green, “unstimulated MDM”), (iii) capsular fibroblasts co-cultured with MerTK^{high} (Dexamethasone stimulated) MDM (blue, “MerTK^{high} MDM”) and (iv) capsular fibroblasts co-cultured with MerTK^{low} (LPS stimulated) MDMs (purple, “MerTK^{low} MDM”) (principle components analysis; first 2 principle components shown). For these experiments capsular fibroblasts were derived from frozen shoulder patients (n=3 donors). Co-culture experiments were performed for 48 hours. Fibroblasts were isolated by FACS sorting prior to transcriptional profiling by bulk RNA-sequencing.

(B) The heatmap shows genes found to have significant variation in expression between the untreated capsular fibroblasts, the capsular fibroblasts co-cultured with MerTK^{high}-MDM and the capsular fibroblasts co-cultured with MerTK^{low} MDMs (DESeq2; LRT test; BH adjusted P<0.05). Rows were clustered into four groups by hierarchical clustering using Kendall pair-wise correlation statistics, followed by tree-cut using the divisive coefficient (DEGreports, http://lpantano.github.io/DEGreport/).

(C) The bar chart shows the change in expression weight for the given predicted cell-cell interactions between MERTK+LYVE1high and DKK3+FMOD+, and MERTK+LYVE1high and ACAN+POSTN+ in frozen shoulder relative to comparator patient tissues.

(D) The Sankey plot shows the mapping between the clusters of macrophages identified in the adult shoulder capsule tissues (Figure 1) (left) and labels transferred from the STM sub-populations reported by Alivernini *et al*. (2020) (right) (scArches analysis).

(E) Box plot shows cassette score of 224 marker genes of MERTK+LYVE1^{high} cluster within shoulder capsule and knee synovial (FOLR2+LYVE1^{high}) macrophages. (Logistic regression, *P*=1.50 x10$^{-139.}$, Wald test).

(F) Scatter plot shows a correlation of 0.21 (Wilcoxon test *P*=2.42 x10^{-5}) in log2 fold changes between healthy and Frozen Shoulder of the shoulder capsule and healthy and remission RA of the knee synovium.

(G) The box plots show statistically significant qualitative differences in gene regulation direction in diseased vs healthy tissue in the frozen shoulder capsule (this study) compared to RA knee synovial tissue samples (Alivernini *et al*., (2020)) in the MERTK+ macrophage populations (BH adjusted *P*≤0.05). The MERTK+ macrophages in the Alivernini *et al.* 2020 samples were inferred by label transfer. Relative to healthy controls, the blue box indicates genes that were upregulated in diseased knee RA synovial tissues and down regulated in diseased frozen shoulder tissues. The red box shows genes with the opposite pattern of expression (Supplementary Data 3). Bar shows median values.

Figure S9

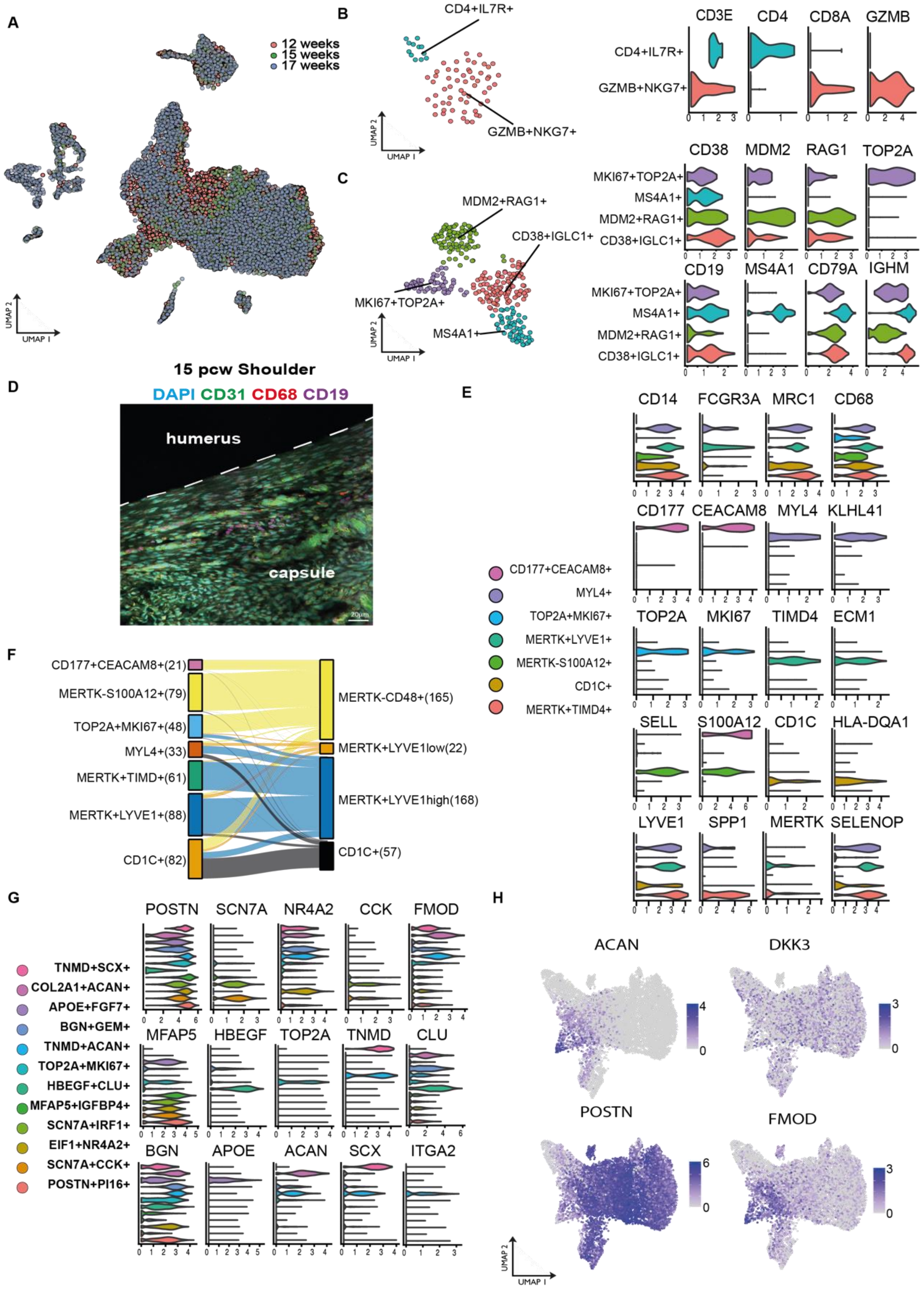

S9. Single cell atlas of the developing shoulder joint: supplementary data

- **(A)** UMAP of all the cells found to be present the shoulder joint during foetal development (as shown in Figure 6A) coloured by developmental timepoint.
- **(B)** UMAP (res=0.3) shows identified foetal T cell populations in the developing shoulder joint. Merged data from 12, 15 and 17 pcw are shown. Violin plots represent log-normalized expression values of top DEGs of T cell clusters.
- **(C)** UMAP (res=0.3) shows foetal B cell clusters comprised of progenitor and differentiated B cell populations. Violin plots represent log-normalized expression values of top marker genes of B cell clusters.
- **(D)** Representative confocal immunofluorescent image shows immunostaining for CD31 (green) CD68 (red) and CD19 (violet) in the developing shoulder capsule at 15pcw. B cells are predominantly contained within blood vessels. Nuclear counterstain is POPO1. Scale bar, 20 μm.
- **(E)** Violin plots represent log-normalized expression values of top marker genes of foetal myeloid clusters.

(F) The Sankey plots shows the mapping between the identified foetal myeloid cell clusters (Figure 6B) left and labels transferred from the adult shoulder capsule myeloid populations (Figure 1G) (right) (scArches analysis).

(G) Violin plots represent log-normalized expression values of top marker genes of foetal fibroblast clusters.

(H) Feature plots showing expression of markers of major adult fibroblast populations in adult shoulder capsule in foetal CD45– populations.

Figure S10

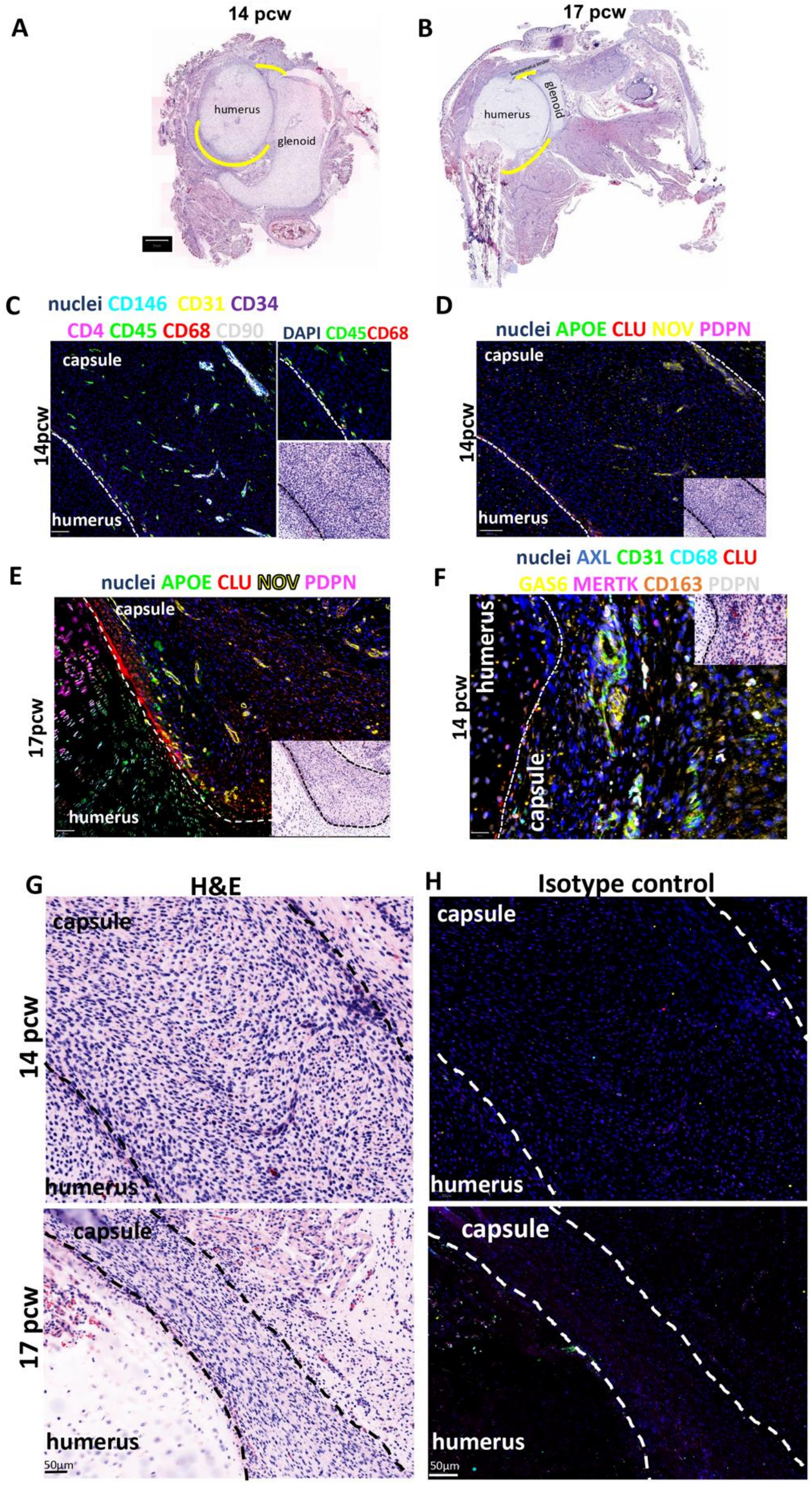

S10. Histology and immunostaining of shoulder joint tissues during embryonic development.

(A) Representative image of coronal H&E stained section of the foetal shoulder joint at 14 post conception weeks (pcw). The location of the shoulder joint capsule is highlighted yellow, scale bar=1mm.

(B) Representative image of sagittal H&E stained section of the foetal shoulder joint at 17 post conception weeks (pcw). The location of the shoulder joint capsule is highlighted yellow, scale bar=1mm.

(C) Representative Cell DIVE and respective H&E stained images of histological sections of the shoulder joint at 14 post conception weeks (pcw) development stage. Section stained for a panel of markers to identify immune cells (CD45, CD68, CD4), endothelial cells (CD31, CD146) and fibroblasts (CD90, CD34). Scale bar=50μm.

(D) 14 pcw shoulder section stained with an extended panel of markers for key fibroblast subsets including PDPN, APOE, CLU, NOV, note CLU+ cells predominate in the capsule lining, NOV+ cells populate the capsule sub-lining Scale bar=50μm.

(E) 17 pcw shoulder section stained with an extended panel of markers for key fibroblast subsets including PDPN, APOE, CLU, NOV, note CLU+ cells predominate in the capsule lining, NOV+ cells populate the capsule sub-lining Scale bar=50μm.

(F) High magnification image showing the cell types identified in developmental shoulder tissues including macrophages (CD68, CD163, MERTK) and fibroblasts (PDPN, CLU, AXL, GAS6) in the capsule lining region at 14 pcw. Scale bar=20μm.

(G) Panel shows representative bright field images of shoulder capsule at 14 and 17 pcw developmental stages stained with isotype control antibodies for mouse, rabbit and goat IgG fractions. Nuclear counterstain is haematoxylin. Scale bar=50μm.

(H) Representative Cell DIVE images of shoulder capsule at 14 and 17 pcw developmental stages stained with isotype control antibodies for mouse, rabbit and goat IgG fractions. Nuclear counterstain is DAPI. Scale bar=50μm.

Supplementary Table 1A. Primary antibodies used for immunostaining of human shoulder capsule tissues.

Epitope	Supplier & Catalogue #	Clone	Isotype	Species	Dilution
Podoplanin (PDPN)	Abcam Ab10288	18H5	IgG$_1$	mouse	1:100
CD90 (THY1)	Abcam Ab181469	7E1B11	IgG1	mouse	1:200
CD34	Abcam Ab54208	9B10D4/4H5E7	IgG2b	mouse	1:200
PDGFRL	Proteintech 16217-1-AP		IgG	rabbit	1:400
CD248	Abcam Ab204914	EPR17081	IgG	rabbit	1:200
FMOD	GeneTex GTX54035		IgG	rabbit	1:300
DKK3	Proteintech 10365-1-AP		IgG	rabbit	1:200
POSTN	Abcam Ab79946		IgG	rabbit	1:200
CTHRC1	Abcam Ab85739		IgG	rabbit	1:200
CHI3L1	Invitrogen MA536131	A3G10	IgG2a	mouse	1:400
ARC	Abcam Ab183183	EPR18950	IgG	rabbit	1:200
ACTA2	Abcam Ab7817	1A4	IgG2a	mouse	1:200
NOTCH3	Abcam Ab23426		IgG	rabbit	1:200
CXCL12	Abcam Ab18919		IgG	rabbit	1:200
PTGDS	Abcam Ab182141	EP12357	IgG	rabbit	1:300
MFAP5	Abcam Ab171737	EPCSUR1	IgG	rabbit	1:200
CLIC5	Biorbyt orb95213		IgG2a	mouse	1:200
PRG4	Abcam Ab28484		IgG	rabbit	1:300
HBEGF	Biorbyt orb539786		IgG	rabbit	1:200
GAS6	Proteintech 13795-1-AP		IgG	rabbit	1:400
PROS1	Proteintech 16910-1-AP		IgG	rabbit	1:200
AXL	Biorbyt orb95121		IgG1	mouse	1:200
CD68	Agilent M0814	KP1	IgG1	mouse	1:400
CD14	Proteintech 17000-1AP		IgG	rabbit	1:200
MERTK	Abcam Ab52968	Y323	IgG	rabbit	1:200
MERTK	Invitrogen 14-9053	A3KCAT	IgG1	mouse	1:400
TREM2	Proteintech 27599-1-AP		IgG	rabbit	1:400
FOLR2	Invitrogen MA526933	OT14G6	IgG1	mouse	1:200
CD48	Biorbyt orb432138		IgG	rabbit	1:200
CD206 (MRC1)	Abcam Ab117644	5C11	IgG1	mouse	1:200

CD163	LS-Biosciences LS_B10966	34B	$IgG2_a$	mouse	1:150
CD83	Abcam Ab205343			rabbit	1:200
ICAM1	Abcam Ab2213	MEM-111	IgG2a	mouse	1:200
FCGR3A	Abcam Ab183354	SP175	IgG	rabbit	1:200
MARCO	Atlas Antibodies HPA063793		IgG	rabbit	1:300
LYVE-1	Atlas Antibodies HPA042953		IgG	rabbit	1:400
PTGS2	LS-Bio LSC339544	OTI10H5	IgG2a	mouse	1:200
S100A8	Abcam Ab 180735		IgG	rabbit	1:200
AREG	Proteintech 66433-1		IgG1	mouse	1:600
SERPINB2	Abcam Ab137588		IgG	rabbit	1:300
IL1R1	Abcam Ab190078		IgG	rabbit	1:200
ERV1	Abcam Ab168097	1A7	IgG2b	mouse	1:200
FPR2/ALX	Abcam Ab26316	GM1D6	IgG1	mouse	1:200
CD3	BioLegend 300402	UCHT1	IgG1	mouse	1:150
Ki67	LSBio LS-C336803	OTI18G5	IgG1	mouse	1:200

Supplementary Table 1B. Secondary/Isotype antibodies used for immunostaining of human shoulder capsule tissues.

Antibody	Isotype	Supplier & Catalogue #	Dilution
Goat Anti-mouse FITC	IgG1	Southern Biotech 1070-02	1:200
Goat Anti-mouse AF568	IgG2a	Invitrogen A21134	1:200
Goat Anti-mouse AF568	IgG2b	Invitrogen A21144	1:200
Goat Anti-rabbit AF633	IgG	Invitrogen A21070	1:200
FLEX Universal Negative Control Mouse	IgG1, IgG2a, IgG2b, IgG3 & IgM	Dako IR750	Ready to use
FLEX Universal Negative Control Rabbit	IgG	Dako IS600	Ready to use

Supplementary Table 2. Antibodies used for Chipcytometry staining.

Epitope	Label	Clone	Isotype	Supplier & catalogue #	Dilution	Stock conc µg/mL	Working conc µg/mL
CD127	PE	REA614	Recombinant human IgG1	Miltenyi 130-113-414	1:50	150	3.0
CD161	PE	REA631	Recombinant human IgG1	Miltenyi 130-113-596	1:100	37.5	0.38
CD18	PE	7E4	IgG1	Beckton Coulter IM1570U	1:100	7.0	0.07
CD2	PE	RPA-2.10	IgG1,κ	BioLegend 300207	1:500	25	0.13
CD3	PerCP	UCHT1	IgG1,κ	BioLegend 300428	1:500	100	0.2
CD31	PerCP/Cy5.5	WM59	IgG1,κ	BioLegend 303132	1:1000	200	0.2
CD4	PE	RPA-T4	IgG1,κ	BioLegend 300508	1:1000	100	0.1
CD45	FITC	H130	IgG1,κ	BioLegend 304038	1:1000	200	0.2
CD5	PE	UCHT2	IgG2,κ	BioLegend 300607	1:500	100	0.2
CD8	PerCP/Cy5.5	SK1	IgG1,κ	BioLegend 344710	1:150	50	0.33
FAP	PE	427819	IgG1	R&D FAB3715P	1:50	10	0.2
GZMB	PE	QA16A02	IgG1,κ	BioLegend 372208	1:100	100	1.0
GZMK	PE	GM26E7	IgG1,κ	BioLegend 370512	1:100	25	0.25

Supplementary Table 3A. List of primary antibodies used for Cell DIVE multiplexing.

Epitope	Secondaries	Clone	Isotype	Species	Supplier	Catalogue #	Lot #	Concentration
APOE	-	EP1374Y	IgG	Rabbit	Abcam	ab196463	GR3375517-2	10 µg/ml
AXL	-	Polyclonal	IgG	Rabbit	Biossusa	bs-5180R-A555	BA08303547	10 µg/ml
CD3	-	SP162	IgG	Rabbit	Abcam	ab245731	GR3389650-2	10 µg/ml
CD4	-	EPR6855	IgG	Rabbit	Abcam	ab280849	GR3388856-2	10 µg/ml
CD8	-	C8/144B	IgG1	Mouse	Biolegend	372902	B298974	10 µg/ml
CD19	-	EPR5906	IgG	Rabbit	Abcam	ab196515	GR3407141-1	10 µg/ml
CD31	-	C31.3<br>JC/70A	IgG1κ	Mouse	Novus	NBP2-34578AF647	D125818	10 µg/ml
CD34	-	EP373Y	IgG	Rabbit	Abcam	ab195013	GR3342298-3	10 µg/ml
CD45	-	2D1	IgG2a	Mouse	Biolegend	368538	B270005	10 µg/ml
CD55	Goat AF488	Polyclonal	IgG	Goat	R&D	AF2009	KGJ0116111	10 µg/ml
CD68	-	EPR20545	IgG	Rabbit	Abcam	ab280860	GR3448209-1	10 µg/ml
CD90	-	EPR3132	IgG	Rabbit	Abcam	ab181885	GR3372473-2	10 µg/ml
CD146	-	EPR3208	IgG	Rabbit	Abcam	ab196448	1038616-1	10 µg/ml
CD163	-	EDHu-1	IgG1	Mouse	Novus	NB110-40686	149022B-<br>121120-AF647	10 µg/ml
CD206	-	C-10	IgG2a	Mouse	Santa Cruz	sc-376232	E2120	5 µg/ml

CLU/Clusterin	Mouse AF555	350227	IgG2b	Mouse	R&D	MAB2937	YIB0219121	5 µg/ml
DKK3	Rabbit AF555	Polyclonal	IgG	Rabbit	Proteintech	10365-1-AP	00047313	1:150 Dilution
FMOD	Rabbit AF647	Polyclonal	IgG	Rabbit	GeneTex	GTX54035	822101963	1:200 Dilution
GAS6	Rabbit AF647	Polyclonal	IgG	Rabbit	Proteintech	13795-1-AP	00068713	1:200 Dilution
LYVE1	-	EPR21857	IgG	Rabbit	Abcam	ab232935	GR3400649-4	10 µg/ml
MERTK	-	Y323	IgG	Rabbit	Abcam	ab52968	GR208748-25	10 µg/ml
NOV	Goat AF647	Polyclonal	IgG	Goat	R&D	AF1640	JKB0218081	10 µg/ml
PDPN	-	D2-40	IgG1κ	Mouse	BioLegend	916610	B384225	10 µg/ml
POSTN	-	EPR20806	IgG	Rabbit	Abcam	ab227049	GR3281155-6	10 µg/ml
TNMD	Rabbit AF488	Polyclonal	IgG	Rabbit	Abcam	ab203676	GR3263244-7	10 µg/ml

Supplementary Table 3B: List of Secondary/Isotype antibodies used for Cell DIVE multiplexing, all reagents were used at 1:500 dilution.

Antibody	Clone	Isotype	Supplier	Catalogue #	Lot #
Donkey Anti-Rabbit AF488	Polyclonal	IgG	Thermo	A21206	2256732
Donkey Anti-Rabbit AF647	Polyclonal	IgG	Thermo	A31573	2181018
Donkey Anti-Rabbit AF555	Polyclonal	IgG	Thermo	A31572	2180682
Donkey Anti-Mouse AF555	Polyclonal	IgG	Thermo	A31570	2253917
Donkey Anti-Goat AF488	Polyclonal	IgG	Abcam	ab150129	GR3375501-2
Donkey Anti-Goat AF555	Polyclonal	IgG	Abcam	ab150130	GR3374229-3
Donkey Anti-Goat AF647	Polyclonal	IgG	Abcam	ab150131	GR3246238-8
Donkey Anti-Rat AF647	Polyclonal	IgG	Abcam	ab150155	GR3379593-2
Rabbit Isotype	Polyclonal	IgG	Biossusa	bs-0295P	AI06186209
Mouse Isotype	Polyclonal	IgG	Biossusa	bs-0295P	AH12061759
Goat Isotype	Polyclonal	IgG	Biossusa	bs-0294P	AE09281424
Rat Isotype	eBR2a	IgG2a	Thermo	16-4321-81	2251911
Rabbit Isotype AF488	Polyclonal	IgG	Biossusa	bs-0295P	AG07067252
Rabbit Isotype AF555	Polyclonal	IgG	Biossusa	bs-0295P	AH07185432

Rabbit Isotype AF647	Polyclonal	IgG	Biossusa	bs-0295P	AI12168487
Mouse Isotype AF488	Polyclonal	IgG	Biossusa	bs-0296P	AE121916
Mouse Isotype AF647	Polyclonal	IgG	Biossusa	bs-0296P	AF08076690

Supplementary Table 4A. Antibodies used to characterize MDMs after dexamethasone or LPS treatments to induce a MERTK^{high} or MERTK^{low} phenotype respectively.

Epitope	Label	Clone	Supplier	Clone	Catalogue #	Dilution
MERTK	APC	590H11G1E3	Biolegend	590H11G1E3	367612	1:100
CD163	PE-Cy7	RM3/1	Biolegend	RM3/1	326514	1:100
CD206	PE	15-2	Biolegend	15-2	321106	1:100
TREM2	AF700	237920	R&D systems	237920	FAB17291N	1:100
CD68	BV785	Y1/82A	Biolegend	Y1/82A	333826	1:100
CD14	BV510	M5E2	Biolegend	M5E2	301842	1:100
LYVE-1	AF488	Polyclonal	Invitrogen	Polyclonal	PA5-22783	1:100
CD48	PerCP-Cy5.5	BJ40	Biolegend	BJ40	336716	1:100
Live/dead	APC-Cy7		BD Biosciences		565388	1:500
Fc block	-		Biolegend		422301	1:100

Supplementary Table 4B. Antibodies used for cell sorting of fibroblasts and monocyte derived macrophages.

Epitope	Label	Clone	Supplier	Catalogue #	Lot number	Dilution
PDPN	AF488	NC-08	Biolegend	337006	B172519	1:200
CD90	PE	5E10	Biolegend	328110	B301003	1:200
CD45	BV605	HI30	Biolegend	304042	B181419	1:200
CD14	BV510	M5E2	Biolegend	301842	B313091	1:200
CD31	AF700	WM59	Biolegend	303134	B274121	1:200
Live/dead	APC-Cy7		BD Biosciences	565388	0167390	1:500
