# Supplementary material for: A single cell atlas of frozen shoulder capsule identifies features associated with inflammatory fibrosis resolution
Source: Nat Commun. 2024 Feb 19;15:1394. doi: 10.1038/s41467-024-45341-9 (PMC10876649; doi:10.1038/s41467-024-45341-9)
Supplement: Supplementary file 3 — Description of Additional Supplementary Files [file 41467_2024_45341_MOESM3_ESM.pdf]

Description of Additional Supplementary Files

File Name: Supplementary Data 1
Description: Human tissue samples used for the study

File Name: Supplementary Data 2
Description: Adult shoulder capsule atlas cluster markers summary

File Name: Supplementary Data 3
Description: Myeloid DEG's frozen shoulder and comparator expression

File Name: Supplementary Data 4
Description: Fibroblast DEG's frozen shoulder and comparator expression

File Name: Supplementary Data 5
Description: DEGs in fibroblasts co-incubated in $MerTK^{high}$ vs $MERTK^{low}$ MDMs

File Name: Supplementary Data 6
Description: Foetal shoulder joint atlas cluster markers summary
