# Supplementary material for: A single cell atlas of frozen shoulder capsule identifies features associated with inflammatory fibrosis resolution
Source: Nat Commun. 2024 Feb 19;15:1394. doi: 10.1038/s41467-024-45341-9 (PMC10876649; doi:10.1038/s41467-024-45341-9)
Supplement: Supplementary file 10 — Reporting Summary [file 41467_2024_45341_MOESM10_ESM.pdf]

nature portfolio

Corresponding author(s): Prof Stephanie G Dakin
Dr Michael TH Ng

Last updated by author(s): Jan 11, 2024

Reporting Summary

Nature Portfolio wishes to improve the reproducibility of the work that we publish. This form provides structure for consistency and transparency in reporting. For further information on Nature Portfolio policies, see our Editorial Policies and the Editorial Policy Checklist.

Statistics

For all statistical analyses, confirm that the following items are present in the figure legend, table legend, main text, or Methods section.

n/a	Confirmed	
☐	☒	The exact sample size (*n*) for each experimental group/condition, given as a discrete number and unit of measurement
☐	☒	A statement on whether measurements were taken from distinct samples or whether the same sample was measured repeatedly
☐	☒	The statistical test(s) used AND whether they are one- or two-sided<br>*Only common tests should be described solely by name; describe more complex techniques in the Methods section.*
☐	☒	A description of all covariates tested
☐	☒	A description of any assumptions or corrections, such as tests of normality and adjustment for multiple comparisons
☐	☒	A full description of the statistical parameters including central tendency (e.g. means) or other basic estimates (e.g. regression coefficient) AND variation (e.g. standard deviation) or associated estimates of uncertainty (e.g. confidence intervals)
☐	☒	For null hypothesis testing, the test statistic (e.g. *F*, *t*, *r*) with confidence intervals, effect sizes, degrees of freedom and *P* value noted<br>*Give P values as exact values whenever suitable.*
☒	☐	For Bayesian analysis, information on the choice of priors and Markov chain Monte Carlo settings
☒	☐	For hierarchical and complex designs, identification of the appropriate level for tests and full reporting of outcomes
☒	☐	Estimates of effect sizes (e.g. Cohen's *d*, Pearson's *r*), indicating how they were calculated

Our web collection on statistics for biologists contains articles on many of the points above.

Software and code

Policy information about availability of computer code

Data collection	Acquisition of multiplex immunofluorescent images was performed with a Zeiss LSM 710 confocal microscope (adult shoulder capsule tissues) or GE Cell DIVE system (adult & foetal shoulder capsule tissues). Two-dimensional image reconstructions were created using ZEN Black software (Zeiss version 11.0.3.190).<br>Viable cells liberated from tissue digests for scRNAseq were sorted by flow cytometry using a SONY SH800 cell sorter. Microcultures of MDMs and patient-derived fibroblasts were sorted using a BD FACSAria III high speed cell sorter with FACSDiva (v8.0.1).<br>scRNAseq of adult and foetal tissue digests was performed using scRNAseq 10xGenomics<br>Sequencing data was generated using Illumina NovaSeq6000. Single cell gene expression matrix was generated using 10x Cell Ranger (version 6.1) and bulk gene expression matrix was generated using Salmon (version 1.5.2). CellDive images were generated using ImageApp (version 1.0). Images of T cell markers in frozen shoulder patient tissues were generated using the Zellscanner App (version 19.08.2020, Zellkraftwerk Gmbh) images were edited using FIJI (version 2.3.0).
Data analysis	We used Qupath (version 0.3) for image visualisation of the Cell DIVE data.<br>We used MATLAB (version R2023a) and CellProfiler (version 4.2.5) to quantify the proportions of major cell types comprising adult shoulder capsule tissues.<br>For analysing FACS data, we used Flowjo (version 10) and GraphPad Prism (version 9.2.0) for statistical analysis.<br>For analysing single cell sequencing data, ambient background RNA was removed using CellBender (version 0.2.0). Doublets were determined using Scrublet (version 0.2.3) The count matrix was transformed into a Seurat object (version 4.0.4) . Latent space was corrected using Harmony (version 0.1.0). Downstream analyses including UMAP generation and neighbour clustering were performed using the Cellhub workflow (https://github.com/sansomlab/cellhub). Pathway analysis was performed using gsfisher (https://github.com/sansomlab/gsfisher) and Clusterprofiler (version 4.2.2). Differential expression analysis was performed using DESeq2 (version 1.26.0).<br>Single-cell gene regulatory network analysis of the fibroblasts was performed using pySCENIC (version 0.11.2) and the elicited biomolecular

interaction networks and regulons were visualized using iRegulon and Cytoscape (version 3.9.1)
Deconvolution was performed using MuSiC (version 0.2.0) on objects created using BisqueRNA (version 1.0.5) and BioBase (version 2.54.0).
For the bulk sequencing data, we used Limma (version 3.50.0) for batch effect correction and DESeq2 (version 1.34.0) for differential expression analysis.

CODE AVAILABILITY:
The code used to perform quantitative analysis of Cell DIVE images for Figure S1E is available at Zenodo (https://zenodo.org/record/8138743).
A lightweight interactive visualisation tool for the adult shoulder capsule scRNAseq data is available on https://capsule.ndorms.ox.ac.uk

For manuscripts utilizing custom algorithms or software that are central to the research but not yet described in published literature, software must be made available to editors and reviewers. We strongly encourage code deposition in a community repository (e.g. GitHub). See the Nature Portfolio guidelines for submitting code & software for further information.

Data

Policy information about availability of data

All manuscripts must include a data availability statement. This statement should provide the following information, where applicable:

- Accession codes, unique identifiers, or web links for publicly available datasets
- A description of any restrictions on data availability
- For clinical datasets or third party data, please ensure that the statement adheres to our policy

DATA AVAILABILITY:
The single cell-RNAseq data generated in this study have been deposited in the ENA database under accession code ERP143358 (https://www.ebi.ac.uk/ena/browser/view/PRJEB58305). The bulk-RNAseq data generated in this study have been deposited in the ENA database under accession code ERP143359 (https://www.ebi.ac.uk/ena/browser/view/PRJEB58306). Human reference genome GRCh38 (GENCODE v32 / Ensembl 98) was used in the alignment of the bulk and single cell transcriptomic data. Source data of the figures are provided with this manuscript.

Research involving human participants, their data, or biological material

Policy information about studies with human participants or human data. See also policy information about sex, gender (identity/presentation), and sexual orientation and race, ethnicity and racism.

Reporting on sex and gender	Demographics and clinical characteristics of patient cohorts used in this study are described in Methods and Supplementary Table 1. Adult tissues used for this study were collected from both male and female donors with adequate representation of each sex present in comparator and frozen shoulder patient cohorts described in Table 1. Foetal tissues used for this study were also collected from male and female donors also shown in Table 1.
Reporting on race, ethnicity, or other socially relevant groupings	We collected musculoskeletal tissues from male and female patients from ethnically diverse backgrounds, ensuring representation of tissues are collected from Asian, Black, mixed, white and other ethnic groups. We included male and female patients over 18 and under 80 years of age. We controlled for confounding variables by excluding patients with immune-mediated inflammatory joint disease. We also excluded frozen shoulder patients who had received a corticosteroid injection into the shoulder capsule less than 3 months prior arthroscopic capsular release surgery.
Population characteristics	Demographics and clinical characteristics of patient cohorts used in this study are described in Methods and Supplementary Table 1. The ages of adult shoulder capsule tissue donors are displayed in Table 1 (rounded to nearest 5 years). The ages of foetal donor tissues were 12, 15 and 17 post conception weeks.
Recruitment	All recruited adult patients were undergoing elective shoulder surgery. Patients were given written information and time to consider the information prior to their surgery to consent for the study. Participants were given the opportunity to have any queries answered by a member of the local research team prior to obtaining full, written and informed consent. Written Informed Consent was be obtained by means of participant dated signature and dated signature of the person who obtained informed consent. Comparator adult shoulder capsule tissues were collected from patients undergoing elective shoulder stabilisation or shoulder arthroplasty surgical procedures. Frozen shoulder patient tissues were collected from patients with advanced-stage disease (≥12 months symptom duration, Table 1) undergoing surgical arthroscopic capsular release. There was no bias in recruitment and this was not a clinical trial.
Ethics oversight	Human adult tissues: The University of Oxford Research Governance & Ethics Assurance approved the protocol for this study. Ethical approval was granted by the local research ethics committee (ICECAP study REC reference 18/SC0649, IRAS project ID 257757) and the Oxford Musculoskeletal Biobank (19/SC/0134). Full informed consent according to the Declaration of Helsinki was obtained from all patients.<br>Human foetal tissues: Samples were provided voluntarily with appropriate informed consent, the HDBR tissue bank operates under Research Ethics Committee approvals 18/NE/0290 (Newcastle upon Tyne) and 18/LO/0822 (London).

Note that full information on the approval of the study protocol must also be provided in the manuscript.

Field-specific reporting

Please select the one below that is the best fit for your research. If you are not sure, read the appropriate sections before making your selection.

☒ Life sciences ☐ Behavioural & social sciences ☐ Ecological, evolutionary & environmental sciences

For a reference copy of the document with all sections, see nature.com/documents/nr-reporting-summary-flat.pdf

Life sciences study design

All studies must disclose on these points even when the disclosure is negative.

Sample size

No statistical methods were used to predetermine sample size for scRNAseq (n=3-6 patients/per group). We used a pragmatic choice of sample numbers based on the numbers used in the emerging clinical scRNAseq data set literature, for example, Croft et al Nature (2019), Alivernini et al, Nature Medicine (2020). All conclusions derived from scRNAseq data were subject to subsequent robust validation by IHC and IF.
Collection of adult shoulder capsule tissues: Comparator and frozen shoulder adult tissue biopsies were collected from the rotator interval of the shoulder (glenohumeral) joint capsule. Comparator tissues were collected from patients undergoing elective shoulder stabilisation (n=8) or shoulder arthroplasty surgical procedures (n=12). Frozen shoulder tissues were collected from patients with advanced-stage disease (≥12 months symptom duration) undergoing surgical arthroscopic capsular release (n=15).
For single cell RNA-seq experiments on adult tissues, 10 shoulder capsule tissues were collected including 6 comparator and 4 frozen shoulder donor samples. For single cell RNA-seq experiments on foetal shoulder tissues, 3 samples were processed from 12, 15 and 17 post conception week ages.
For quantitative analysis of immunopositive staining for markers of macrophages, fibroblast activation and matrix proteins, tissue sections from a minimum of n=5 comparator and n=5 frozen shoulder donors was used.
To characterize the blood cone derived macrophages after stimulating with Dexamethasone or LPS to induce MerTKhigh and MerTKlow macrophage phenotypes respectively, protein expression was measured by flow cytometric analysis from 4 different donors in independent experiments.
For functional experiments using microcultures of patient-derived cells, cells from n=3 frozen shoulder donors and n=3 non-clinical blood cones were performed in 3 independent experiments.

Data exclusions

For the single cell data analysis, we removed ambient background RNA, cells that had abnormally low or high gene counts (<300 or >6500 n_Feature) and high mitochondrial gene expression (>10% mitochondrial genes). After clustering the retained cells, we further removed clusters of cells with low UMI (nCount <10000) and gene count (n_Features<1000), and a high level of expression in hemoglobin markers (HBA, HBB, HBG). We also excluded clusters of cells that consisted mainly doublets (score >=0.4). For differential expression analysis, we analysed genes that were detectable (count ≥10) in at least a reasonable subset of samples (n >= x where x is the sample count of the minor condition).

For the bulk RNA data analysis, we applied the same DEG filtering strategy described above but modified the definition of reasonable subset of sample (n>=x where x is the sample count of the biological replicates) to fit the experimental plan.

Replication

For scRNAseq experiments, cell types detected in shoulder capsule tissues were reproducible across donors.
All in vitro experiments were repeated 3 times and showed similar data.

Randomization

It was not possible to randomize adult patient tissue samples as all patients were undergoing elective surgery. We excluded patients with immune-mediated inflammatory joint disease. We also excluded frozen shoulder patients who had received a corticosteroid injection into the shoulder capsule less than 3 months prior arthroscopic capsular release surgery.

Blinding

For single cell RNAseq and bulk RNAseq data, investigators were not blinded to allocation during experiments and outcome assessment.
Quantitative analysis of histological capsule tissues sections was performed by a single investigator who was blinded to the health status of the shoulder capsule tissue.

Reporting for specific materials, systems and methods

We require information from authors about some types of materials, experimental systems and methods used in many studies. Here, indicate whether each material, system or method listed is relevant to your study. If you are not sure if a list item applies to your research, read the appropriate section before selecting a response.

Materials & experimental systems

n/a	Involved in the study	
☐	☒	Antibodies
☒	☐	Eukaryotic cell lines
☒	☐	Palaeontology and archaeology
☒	☐	Animals and other organisms
☒	☐	Clinical data
☒	☐	Dual use research of concern
☒	☐	Plants

Methods

n/a	Involved in the study	
☒	☐	ChIP-seq
☐	☒	Flow cytometry
☒	☐	MRI-based neuroimaging

Antibodies

Antibodies used

Primary antibodies used for IHC / IF on FFPE adult shoulder capsule tissue sections (also shown in Supplementary Table 1A)
IgG1 mouse anti-human podoplanin (clone 18H5, Abcam Ab10288, 1:100 dilution)
IgG1 mouse anti-human CD90 (clone 7E1B11, Abcam, Ab181469, 1:200 dilution)

IgG2b mouse anti-human CD34 (clone 9B10D4/4H5E7 Abcam, Ab54208 1:200 dilution)
Polyclonal rabbit anti-human PDGFRL (Proteintech 16217-1-AP, 1:400 dilution)
Monoclonal rabbit anti-human CD248 (clone EPR17081, Abcam, Ab204914, 1:200 dilution)
Polyclonal rabbit anti-human FMOD (GeneTex, GTX54035, 1:300 dilution)
Polyclonal rabbit anti-human DKK3 (Proteintech 10365-1-AP, 1:200 dilution)
Polyclonal rabbit anti-human POSTN (Abcam, Ab79946, 1:200 dilution)
Polyclonal rabbit anti-human CTHRC1 (Abcam, Ab85739, 1:200 dilution)
IgG2a mouse anti-human CHI3L1 (clone A3G10, Invitrogen, MA536131, 1:400 dilution)
Monoclonal rabbit anti-human ARC (clone EPR18950, Abcam, Ab183183, 1:200 dilution)
IgG2a mouse anti-human ACTA2 (clone 1A4, Abcam Ab7817, 1:200 dilution)
Polyclonal rabbit anti-human NOTCH3 (Abcam Ab23426, 1:200 dilution)
Polyclonal rabbit anti-human CXCL12 (Abcam Ab18919, 1:200 dilution)
Monoclonal rabbit anti-human PTGDS (clone EP12357, Abcam, Ab182141, 1:300 dilution)
Monoclonal rabbit anti-human MFAP5 (MAGP2) (clone EPCSUR1, Abcam, Ab171737, 1:200 dilution)
IgG2a mouse anti-human CLIC5 (Biorbyt, orb95213, 1:200 dilution)
Polyclonal rabbit anti-human PRG4 (Abcam Ab28484, 1:300 dilution)
Polyclonal rabbit anti-human HBEGF (Biorbyt, orb539786, 1:200 dilution)
Polyclonal rabbit anti-human GAS6 (Proteintech, 13795-1-AP, 1:400 dilution)
Polyclonal rabbit anti-human PROS1 (Proteintech16910-1-AP, 1:200 dilution)
IgG1 mouse anti-human AXL (Biorbyt, orb95121, 1:200 dilution)
IgG1 mouse anti-human CD68 (clone KP1, Agilent, M0814, 1:400 dilution)
Polyclonal rabbit anti-human CD14 (Proteintech, 17000-AP, 1:200 dilution)
Monoclonal rabbit anti-human MERTK (clone Y323, Abcam, Ab52968, 1:200 dilution)
IgG1 mouse anti-human MERTK (clone A3KCAT, Invitrogen,14-9053, 1:400 dilution)
Polyclonal rabbit anti-human TREM2 (Proteintech 27599-1-AP, 1:400 dilution)
IgG1 mouse anti-human FOLR2 (clone OT14G6, Invitrogen, MA526933 1:200 dilution)
Polyclonal rabbit anti-human CD48 (Biorbyt orb432138, 1:200 dilution)
IgG1 mouse anti-human CD206 (clone 5C11, Abcam Ab117644, 1:200 dilution)
IgG2a mouse anti-human CD163 (clone 34B, LS-Biosciences, LS_B10966, 1:150 dilution)
Polyclonal rabbit anti-human CD83 (Abcam Ab205343, 1:200 dilution)
IgG2a mouse anti-human ICAM1 (clone MEM111, Abcam Ab2213 1:200 dilution)
Monoclonal rabbit anti-human FCGR3A (clone SP175, Abcam Ab183354, 1:200 dilution)
Polyclonal rabbit anti-human MARCO (Atlas Antibodies, HPA063793, 1:300 dilution)
Polyclonal rabbit anti-human LYVE1 (Atlas Antibodies, HPA042953, 1:400 dilution)
IgG2a mouse anti-human PTGS2 (clone OTI10H5, LS-Bio LSC339544, 1:200 dilution)
Polyclonal rabbit anti-human S100A8 (Abcam Ab180735, 1:200 dilution)
Polyclonal rabbit anti-human AREG (Proteintech 66433-1, 1:600 dilution)
Polyclonal rabbit anti-human SERPINB2 (Abcam Ab137588, 1:300 dilution)
Polyclonal rabbit anti-human IL1R1 (Abcam Ab190078, 1:200 dilution)
IgG2b mouse anti-human ERV1 (clone 1A7, Abcam Ab168097, 1:200)
IgG1 mouse anti-human FPR2/ALX (clone GM1D6, Abcam Ab26316, 1:200 dilution)
IgG1 mouse anti-human CD3 (clone UCHT1, BioLegend 300402, 1:150 dilution)
IgG1 mouse anti-human Ki67 (clone OTI18G5, LSBio LS-C336803, 1:200 dilution)

Secondary antibodies and isotype controls used for IHC / IF on FFPE adult shoulder capsule tissue sections (also shown in Supplementary Table 1B)
IgG1 goat anti-mouse FITC (Southern Biotech 1070-02, 1:200 dilution)
IgG2a goat anti-mouse Alexa Fluor 568 (Invitrogen, A21134, 1:200 dilution)
IgG2b goat anti-mouse Alexa Fluor 568 (Invitrogen, A21144, 1:200 dilution)
IgG goat anti-rabbit Alexa Fluor 633 (Invitrogen, A21070, 1:200 dilution)
FLEX Universal Negative Control, Mouse IgG1, IgG2a, IgG2b, IgG3 and IgM isotypes Ready-to-Use (Dako IR750)
FLEX Universal Negative Control, Rabbit, Ready-to-Use (Dako IS600)

Chipcytometry antibodies used to stain cryosections of adult shoulder capsule tissue sections (also shown in Supplementary Table 2)
IgG1 PE anti-human CD127 (clone REA614, Miltenyi, 130-113-414, 1:50 dilution)
IgG1 PE anti-human CD161 (clone REA631, Miltenyi, 130-113-596, 1:100 dilution)
IgG1 PE anti-human CD18 (clone 7E4, Beckton Coulter, IM1570U, 1:100 dilution)
IgG1 PE anti-human CD2 (clone RPA-2.10, Biolegend, 300207, 1:500 dilution)
IgG1 PerCP anti-human CD3 (clone UCHT1, Biolegend, 300428, 1:500 dilution)
IgG1 PerCP/Cy5.5 anti-human CD31 (clone WM59, Biolegend, 303132, 1:1000 dilution)
IgG1 PE anti-human CD4 (clone RPA-T4, Biolegend, 300508, 1:1000 dilution)
IgG1 FITC anti-human CD45 (clone H130, Biolegend, 304038, 1:1000 dilution)
IgG2 PE anti-human CD5 (clone UCHT2, Biolegend, 300607, 1:500 dilution)
IgG1 PerCP/Cy5.5 anti-human CD8 (clone SK1, Biolegend 344710, 1:150 dilution)
IgG1 PE anti-human FAP (clone 427819, R&D FAB3715P, 1:50 dilution)
IgG1 PE anti-human GZMB (clone QA16A02, Biolegend 372208, 1:100 dilution)
IgG1 PE anti-human GZMK (clone GM26E7, Biolegend 370512, 1:100 dilution)

Primary antibodies used for IHC / IF for Cell DIVE staining of human shoulder capsule tissue sections (also shown in Supplementary Table 3A)
Monoclonal rabbit anti-human APOE (clone EP1374Y, Abcam ab196463, concentration 10 μg/ml)
Polyclonal rabbit anti-human AXL (Biossusa, bs-5180R-A555, concentration 10 μg/ml)
Monoclonal rabbit anti-human CD3 (clone SP162, Abcam ab245731, concentration 10 μg/ml)
Monoclonal rabbit anti-human CD4 (clone EPR6855, Abcam ab280849, concentration 10 μg/ml)
IgG1 mouse anti-human CD8 (clone C8/144B, Biolegend 372902, concentration 10 μg/ml)
Monoclonal rabbit anti-human CD19 (clone EPR5906, Abcam ab196515, concentration 10 μg/ml)

IgG1 mouse anti-human CD31 (clone c31.3, JC/70A Novus, NBP2-34578AF647, concentration 10 µg/ml)
Monoclonal rabbit anti-human CD34 (clone EP373Y, Abcam, ab195013, concentration 10 µg/ml)
IgG2a mouse anti-human CD45 (clone 2D1, Biolegend 368538, concentration 10 µg/ml)
Polyclonal rabbit anti-human CD55 (R&D AF2009, concentration 10 µg/ml)
Monoclonal rabbit anti-human CD68 (clone EPR20545, Abcam, ab280860, concentration 10 µg/ml)
Monoclonal rabbit anti-human CD90 (clone EPR3132, Abcam ab181885, concentration 10 µg/ml)
Monoclonal rabbit anti-human CD146 (clone EPR3208, Abcam, ab196448, concentration 10 µg/ml)
IgG1 mouse anti-human CD163 (clone EDHu-1, Novus NB110-40686, concentration 10 µg/ml)
IgG2a mouse anti-human CD206 (clone C10, Santa Cruz sc-376232, concentration 5 µg/ml)
IgG2b mouse anti-human CLU (clone 350227, R&D MAB2937, concentration 5 µg/ml)
Polyclonal rabbit anti-human DKK3 (Proteintech, 10365-1-AP, 1:150 Dilution)
Polyclonal rabbit anti-human FMOD (GeneTex,GTX54035, 1:200 Dilution)
Polyclonal rabbit anti-human GAS6 (Proteintech, 13795-1-AP, 1:200 dilution)
Monoclonal rabbit anti-human LYVE1 (clone EPR21857, Abcam ab232935, concentration 10 µg/ml)
Monoclonal rabbit anti-human MERTK (clone Y323, Abcam ab52968, concentration 10 µg/ml)
Polyclonal rabbit anti-human NOV (R&D AF1640, concentration 10 µg/ml)
IgG1κ mouse anti-human PDPN (clone D2-40, BioLegend 916610, concentration 10 µg/ml)
Monoclonal rabbit anti-human POSTN (clone EPR20806, Abcam ab227049, concentration 10 µg/ml)
Polyclonal rabbit anti-human TNMD (Abcam ab203676, concentration 10 µg/ml)

Secondary antibodies & isotype controls used for IHC / IF for Cell DIVE staining of human shoulder capsule tissue sections (also shown in Supplementary Table 3B)
Polyclonal Donkey Anti-Rabbit AF488 (Thermofisher Scientific A21206, 1:500 dilution)
Polyclonal Donkey Anti-Rabbit AF647 (Thermofisher Scientific A31573, 1:500 dilution)
Polyclonal Donkey Anti-Mouse AF555 (Thermofisher Scientific, A31570, 1:500 dilution)
Polyclonal Donkey Anti-Goat AF488 (Abcam ab150129, 1:500 dilution)
Polyclonal Donkey Anti-Goat AF555 (Abcam ab150130, 1:500 dilution)
Polyclonal Donkey Anti-Goat AF647 (Abcam ab150131, 1:500 dilution)
Polyclonal Donkey Anti-Rat AF647 (Abcam ab150155, 1:500 dilution)
Polyclonal Donkey Anti-Rabbit AF555 (Thermofisher Scientific A31572, 1:500 dilution)
Polyclonal Rabbit Isotype (Biossusa bs-0295P, 1:500 dilution)
Polyclonal Mouse Isotype (Biossusa bs-0295P, 1:500 dilution)
Polyclonal Goat Isotype (Biossusa bs-0294P, 1:500 dilution)
IgG2a Rat Isotype (clone eBR2a, Thermofisher 16-4321-81, 1:500 dilution)
Polyclonal Rabbit Isotype AF488 (Biossusa, bs-0295P, 1:500 dilution)
Polyclonal Rabbit Isotype AF555 (Biossusa bs-0295P, 1:500 dilution)
Polyclonal Rabbit Isotype AF647 (Biossusa bs-0295P, 1:500 dilution)
Polyclonal Mouse Isotype AF488 (Biossusa bs-0296P, 1:500 dilution)
Polyclonal Mouse Isotype AF647 (Biossusa bs-0296P, 1:500 dilution)

Primary antibodies used for flow cytometry of blood-derived macrophages (see also Supplementary Table 4A)
IgG1, κ Mouse anti-human MERTK in APC (clone 590H11G1E3, Biolegend 367612, 1:100 dilution)
IgG1, κ Mouse anti-human CD163 in PE-Cy7 (clone RM3/1, Biolegend 326514, 1:100 dilution)
IgG1, κ Mouse anti-human CD206 in PE (clone 15-2, Biolegend 321106, 1:100 dilution)
IgG2b Rat anti-human TREM2 in AF700 (clone 237920, Biolegend FAB17291N, 1:100 dilution)
IgG2b Mouse anti-human CD68 in BV785 (clone Y1/82A , Biolegend 333826, 1:100 dilution)
IgG2a, κ Mouse anti-human CD14 in BV510 (clone M5E2, Biolegend 301842, 1:100 dilution)
Polyclonal rabbit anti-human LYVE-1 in Dyelight 488 (Invitrogen PA5-22783, 1:100 dilution)
IgG1, κ Mouse anti-human CD48 in PerCP-Cy5.5 (clone BJ40, Biolegend 336716, 1:100 dilution)

Primary antibodies used for sorting primary fibroblast and blood-derived macrophages (see also Supplementary Table 4B)
IgG2a, λ Rat anti-human Podoplanin in AF488 (clone NC-08, Biolegend 337006, 1:200 dilution)
IgG1, κ Mouse anti-human CD90 in PE (clone 5E10, Biolegend 328110, 1:200 dilution)
IgG1, κ Mouse anti-human CD45 in BV605 (clone HI30, Biolegend 304042, 1:200 dilution)
IgG2a, κ Mouse anti-human CD14 in BV510 (clone M5E2, Biolegend 301842, 1:200 dilution)
IgG1, κ Mouse anti-human CD31 in AF700 (clone WM59, Biolegend 303134, 1:200 dilution)

Primary antibodies used for ICC staining of frozen shoulder capsular fibroblasts:
Polyclonal rabbit anti-human POSTN (Abcam ab79946, 1:200 dilution)
IgG2b Mouse anti-human DKK3 (clone 4E6H6, Proteintech, 1:400 dilution)

Secondary antibodies used for ICC staining of frozen shoulder capsular fibroblasts:
IgG2b goat anti-mouse Alexa Fluor 568 (Invitrogen, A21144, 1:200 dilution)
IgG goat anti-rabbit Alexa Fluor 633 (Invitrogen, A21070, 1:200 dilution)
FLEX Universal Negative Control, Mouse IgG1, IgG2a, IgG2b, IgG3 and IgM isotypes Ready-to-Use (Dako IR750)
FLEX Universal Negative Control, Rabbit, Ready-to-Use (Dako IS600)

Validation

All of the antibodies used in this study are commercially available. These reagents have been used in the assays (flow cytometry, immunohistochemistry or immunofluorescence staining) in accordance with manufacturer design detailed in the data-sheets. All primary antibodies have been appropriately validated for use with human tissues and cells by manufacturers for a given application, this information is provided on their website and product information data-sheets easily accessible with the catalogue number. All antibodies described here have been further optimized for an appropriate concentration by testing several dilutions and the positivity of the signal controlled by FMO and isotype controls on human tissues and cells.
Examples of manufacturer specific validation information is listed below:
Abcam: https://www.abcam.com/primary-antibodies/how-we-validate-our-antibodies#IHC%20and%20ICC

GeneTex: https://www.genetex.com/MarketingMaterial/Index/five_pillars#:~:text=GeneTex%27s%20version%20of%20the%20IWGAV,Protein%20Expression%20(Figure%201)
Proteintech: https://www.ptglab.com/support/sirna-knockdown/
BioLegend: https://www.biolegend.com/Files/Images/BioLegend/literature/images/02-0015-01_immunobiology_handbook.pdf

Plants

Seed stocks

N/A

Novel plant genotypes

N/A

Authentication

N/A

Flow Cytometry

Plots

Confirm that:

☒ The axis labels state the marker and fluorochrome used (e.g. CD4-FITC).

☒ The axis scales are clearly visible. Include numbers along axes only for bottom left plot of group (a 'group' is an analysis of identical markers).

☒ All plots are contour plots with outliers or pseudocolor plots.

☒ A numerical value for number of cells or percentage (with statistics) is provided.

Methodology

Sample preparation

Preparation of monocyte derived macrophages from blood cones, preparation of samples for flow cytometry:
Macrophages were derived from leukocyte cones from the NHS blood and Transplant at the JR Hospital in Oxford using PBMC isolation followed by CD14+ without CD16 depletion kit (Stemcell cat. 19058). Macrophages were dissociated from the plate with accutase for 10 minutes, followed by scraping using a cell lifter. The cells were stained with antibodies against the surface markers MERTK, CD163, CD206, TREM2, CD14, LYVE-1 and CD48 (1:100 dilution, details in supplementary) in PBS 10% FBS (inactivated by heating to 56oC for 30mins). In addition, the 7AAD Live/dead stain (BD Biosciences, 565388, 1:500 dilution) and FC block (Biolegend, 422301, 1:100 dilution) was added to the mixture. After 20 minutes of staining at 4 degrees, cells were spun down, and supernatant was discarded before cells were fixed using 4% PFA in PBS at RT for 20 minutes. After spinning and discarding of the supernatant, cells were permeabilized for 15 minutes with 1x BD Perm/Wash™ buffer(BD Biosciences, 554714) and stained with antibody against CD68 for 20 minutes at RT. Cells were washed in permeabilization buffer and resuspended in FACS buffer before running on the LSRII. Data shown in Figure S7C were generated from 4 blood cones in 4 independent experiments.

Sample preparation for sorting frozen shoulder fibroblasts co-incubated with monocyte derived macrophages:
Macrophages were derived from leukocyte cones (n=3) from the NHS blood and Transplant at the JR Hospital in Oxford using PBMC isolation followed by CD14+ without CD16 depletion kit. Capsular fibroblasts were isolated from the rotator interval of tissues collected from frozen shoulder patients (n=3 donors). Fibroblasts and MDMs were co-cultured for 48 hrs in complete media containing 3% FCS prior to harvest and sorting. After 48 hours, cells were incubated with accutase for 10 minutes and vigorously resuspended. Media was added and remaining adherent cells detached by scraping. A single cell suspension was obtained after putting cells through a 70um cell strainer and resuspended in Facs buffer containing 1% BSA 0.1mg/ml DNase in PBS. Cells were treated with Fc block (Biolegend, 422301) stained with antibodies PDPN, CD90, CD45, CD14 (1:200 dilution, Table S13) to distinguish between fibroblasts and MDMs and the 7AAD Live/dead stain was added (BD Biosciences, 565388, 1:500 dilution). Directly co-cultured MDMs and fibroblasts were sorted on a BD Aria III with Diva 8.0.1 software. Fibroblasts (PDPN+CD90+CD31-) and MDMs (CD45+CD14+) were sorted into RLT Lysis buffer (Qiagen). RNA from sorted capsular fibroblasts was extracted using the RNEasy microprep kit (Qiagen) according to the manufacturers protocol.

Instrument

Flow cytometer instrument: LSR II Cat No. 339580 (BD Biosciences)
Cell sort instrument: BD FACSAria III

Software

Software for flow cytometry: BD FACSDiva™, FlowJo_v10.6.1, Graphpad Prism 9.2.0
Software for cell sorting: BD FACSDiva™ 8.0.1

Cell population abundance

Flow cytometry: Purity of the cells are determined by the CD14 positive without CD16 depletion monocyte isolation kit. Total cells recorded ranged between 38.710 and 353.969 cells depending on donor and condition. Live cells would range between 16.955 and 206.751.
Cell sorting: Approximately 300,000 fibroblasts were sorted and used for Bulk RNAsequencing

Gating strategy

Flow cytometry: SSC-A/FSC-A cells, FCS-H/FCS-A singlets, FCS-H/APC-Cy7 gate negative population for live cells, FSC-H/MERTK, FSC-H/CD48. Gate setting for MERTK and CD48 based on unstained sample which was used to define the negative cells populations.
Cell sorting: SSC-A/FSC-A, FSC-H/FSC-W, APC-Cy7/FSC-A negative population for live cells. Fibroblasts were sorted on CD90+PDPN+ CD31- gate. Macrophages were sorted on CD14+CD45+ gate.

☒ Tick this box to confirm that a figure exemplifying the gating strategy is provided in the Supplementary Information.
